# The Effects of Rapamycin on the Intestinal Graft in a Rat Model of Cold Ischemia Perfusion and Preservation

**DOI:** 10.3390/metabo12090794

**Published:** 2022-08-25

**Authors:** Ibitamuno Caleb, Benedek Kasza, Luca Erlitz, Dávid Semjén, Péter Hardi, Lilla Makszin, Szilárd Rendeki, Ildikó Takács, Tibor Nagy, Gábor Jancsó

**Affiliations:** 1 Medical Skills Education and Innovation Centre, University of Pécs Medical School, 7624 Pécs, Hungary; 2 Institute of Pathology, University of Pécs Medical School, 7624 Pécs, Hungary; 3 Institute of Bioanalysis, University of Pécs Medical School, 7624 Pécs, Hungary; 4 Institute of Nutrional Science and Dietetics, Faculty of Health Sciences, University of Pécs, 7621 Pécs, Hungary; 5 Vascular Surgery Clinic, University of Pécs Medical School, 7624 Pécs, Hungary

**Keywords:** small bowel preservation, intestinal mucosa injury, intestinal perfusion, cold ischemia-reperfusion, Rapamycin, preconditioning, autophagy

## Abstract

Attenuating the rheological and structural consequences of intestinal ischemia-reperfusion-injury (IRI) is important in transplant proceedings. Preconditioning is an often-proposed remedy. This technique uses physical or pharmacological methods to manipulate key ischemia pathways, such as oxidation, inflammation, and autophagy, prior to ischemia. This study determined the time-dependent effects of Rapamycin preconditioning on small-bowel grafts undergoing cold ischemia perfusion and preservation. Our main parameters were mucosa and cell injury and autophagy. A total of 30 male Wistar rats were divided into 5 groups: sham, preservation-control, and 3 treated groups (Rapamycin administered either 0, 30, or 60 min prior to perfusion). After perfusion, the intestines were placed in chilled IGL-1 solution for 12 h. Thereafter, they were reperfused. Histology and bioanalysis (LDH and lactate) were used to ascertain intestinal injury while immunohistochemistry was used for measuring changes in autophagy markers (Beclin-1, LC3B, and p62 proteins). The results show no significant difference amongst the groups after vascular perfusion. However, intestinal injury findings and autophagy changes demonstrate that administering Rapamycin 30 min or 60 min prior was protective against adverse cold ischemia and reperfusion of the intestinal graft. These findings show that Rapamycin is protective against cold ischemia of the small intestine, especially when administered 30 min before the onset.

## 1. Introduction

Organ transplant is a well-established therapy for end-stage organ failure. In recent years, patients with intestinal failure can receive bowel implants largely due to advancement in operative techniques and immunosuppressive therapy [1,2,3]. Despite this significant milestone, preservation damage and the resulting ischemia-reperfusion injury (IRI) of the bowel grafts remains a significant problem [1,4,5]. In the process of transplantation, ischemia begins from the point of organ retrieval and lasts until implantation. The lack of blood supply to the organs leads to the disruption of energy production and activates detrimental pathways, such as oxidative stress and apoptosis, the end-result being cellular death [3,6]. In clinical practice, cold perfusion and preservation is often employed to limit this ischemia [2]. Whilst this method seems effective for other organs, it is not specifically tailored for the intestine; significant damage to the intestinal graft still occurs [2,7]. Subepithelial edema of the intestinal villi develops after short periods of cold ischemia. With increasing storage time, this progresses to full villi destruction and crypt damage [2,8]. The alterations present at the end of cold ischemia are worsened by reperfusion, initiating the ischemia-reperfusion cascade [9]. Advanced IRI can cause extensive mucosa damage and impaired barrier function [10]. In turn, these will predispose to bacterial translocation, endotoxemia, and excessive inflammatory response [2,10]. It greatly jeopardizes the outcome of transplantation and may even promote acute rejection [11]. Hence, proactive prevention of ischemic insult should begin around the time of retrieval of the intestinal graft.

Instigating protective molecular responses before the onset of ischemia (preconditioning) either by brief cycles of IR or pharmacological agents has been proven effective in different studies [12,13,14]. However, clinical adaptation of these methods has been limited by the complexities, toxicity, and narrow therapeutic window of some of the compounds [12]. In this study, we approach preconditioning of small bowel grafts using a well-known clinical agent, Rapamycin. Rapamycin, which inhibits the mammalian target of the rapamycin (mTOR) pathway, was originally developed as an anti-fungal agent in the 1970s [15,16]. Based on its good immunosuppressive and antiproliferative effects, it has been adapted severally in the field of organ transplant [17]. When applied during ischemic conditions, the sum of its effects has either been proven as protective, reducing local and systemic effects of ischemia [15,18], or shown to be detrimental, worsening ischemic outcomes [19,20].

Rapamycin’s effects are achieved by the modulation of various pathways that are relevant to ischemia, such as oxidative stress, apoptosis, and autophagy [16,19]. Indeed, Rapamycin acting through mTOR is known to induce autophagy [18,19]. Autophagy, which is a cell degradation-recycling pathway, has gained a lot of attention in the last decade of ischemia-reperfusion research [15,18,21]. This pathway is said to assist in the clearance of damaged organelles and recycles important cell nutrients thereby promoting cell survival [22,23]. Our group has recently illustrated the autophagy pathway in the cold preservation of small bowel grafts [24]. Additionally, recent evidence suggests that autophagy may contribute to the maintenance of intestinal mucosa [25,26].

This study explores the effects of pretreatment with a single dose of Rapamycin on cold ischemic damage of the intestine. We also investigate if the time of Rapamycin administration and the possible resultant effects correlate with the changes in autophagy of the small bowel grafts

## 2. Materials and Methods

### 2.1. Experimental Animals

Healthy male Wistar rats (*n* = 30) weighing between 250–300 g were used for this study. They were housed under standard conditions and fed rat chow and water ad libitum. Food was withdrawn 24 h prior to the experiment. Animals were anesthetized with an intraperitoneal (i.p) mixture of ketamine hydrochloride (0.075 mg/g of body weight) and diazepam (0.075 mg/g of body weight). At the end of the surgical procedure, the animals died due to exsanguination. All procedures were performed in accordance with ethical guidelines (BA02/2000-02/2021) to minimize pain and suffering of the animals.

### 2.2. Intestinal Perfusion and Preservation

After median laparotomy, the intestine was retrogradely perfused via the aorta at 6 mL/min with ice-cold IGL-1 solution (Institute George Lopez-1 solution, Lyon, France). The portal vein was cut to facilitate venous venting. The perfusion lasted until the intestinal gross morphology turned from pink to a pale color, signifying removal of blood from its circulation. At the end of the perfusion, small bowel grafts were resected from the ligament of Treitz and stored in the same solution at 4 °C. After cold storage, grafts were perfused using oxygenated Krebs Henslet buffer solution (KHBS) for 60 min according to an ex vivo method previously described [27]. Intestinal samples, preservation and perfusion fluid were taken at various time points for further analysis.

### 2.3. Experimental Groups

Rats were randomly divided into five groups (*n* = 6/group):

(1) Sham operated: Intestinal samples collected after midline laparotomy;
(2) Preservation control (PC) group: Intestinal samples collected after perfusion, after 12 h of preservation and after reperfusion;
(3) Rapa-0: Rapamycin administered at 0 min before intestinal retrieval; Intestinal samples collected after perfusion, after 12 h of preservation and after reperfusion;
(4) Rapa-30: Rapamycin administered at 30 min before intestinal retrieval; Intestinal samples collected after perfusion, after 12 h of preservation and after reperfusion;
(5) Rapa-60: Rapamycin administered at 60 min before intestinal retrieval; Intestinal samples collected after perfusion, after 12 h of preservation and after reperfusion.

### 2.4. Drug Preparation and Dosage

In total, 2 mg/kg of Rapamycin (Hb2779 Hellobio) was dissolved in 1 mL dimethyl sulfoxide solution (DMSO) [19]. The drug was injected intraperitoneally (i.p) at the different times (0, 30, and 60), corresponding to the experiment group. The preservation control group received same volume of the solvent (DMSO) used.

### 2.5. Small Bowel Injury

Park et al. suggested a scoring system for the microscopic changes of intestinal grafts being preserved [8]. In this study, we adopted this method and evaluated intestinal ischemic injury using this same scoring system. We refer to it in the text as histology injury score (HIS) (See Table 1 below). Differences in LDH as a marker of cell injury has been shown during ischemic events of the intestine. Similarly, changes in lactate are frequently used as marker of cellular hypoxia [14,28]. Therefore, we quantified cell injury by analyzing the amount of lactate and lactate dehydrogenase enzyme (LDH) in the preservation fluid.

### 2.6. Histology (Hematoxylin-Eosin)

Tissues were fixed in 10% neutral buffered formalin and embedded in paraffin. They were cut in 3 μm thick sections and stained with hematoxylin and eosin. Slides were digitized with Mirax scanner and photographs were taken with CaseViewer 2.4 software (3DHISTECH Ltd., Budapest, Hungary). Intestinal mucosa damage was evaluated blindly by two individuals. The degree of injury was determined using the scoring system described by Park et al. [8]. (See Table 1). A minimum of three fields randomly selected from four quadrants of each intestinal sample were evaluated.

Morphometric analysis of total mucosa thickness and villous depth was analyzed using the CaseViewer 2.4 software (3DHISTECH Ltd.). Total mucosa thickness was assessed by measuring the distance between the villus tip to the lamina-muscularis mucosae in at least four axially oriented villi in four quadrants. Crypt depth was determined in at least a total of five axially oriented, open, non-destroyed crypts from three quadrants.

### 2.7. Biochemical Analysis

After 12 h of preservation, fluid samples were obtained and analyzed for the presence of lactate and lactate dehydrogenase (LDH) enzyme. After centrifugation (10 min, room temperature, 1500 rcf), both parameters were quantified using the Cobas integra 400 plus Analyzer (Roche Diagnostics, GmbH, Mannheim, Germany), following the manufacturer’s instructions.

### 2.8. Immunohistochemistry (IHC) Staining for Autophagy Proteins

Beclin-1 protein has been described as a key protein in the initiation of the autophagy complex, while microtubule-associated 1A/1B light chain-3B protein is an essential protein of its elongation step. Both proteins are often used as markers for autophagy upregulation [19,29]. Additionally, the autophagy protein p62/SQSTMI (simply p62) is efficiently degraded by autophagy. Hence, its levels inversely correlates with autophagic activity [19]. The increase in LC3B and Beclin-1, alongside the decrease in p62 protein usually indicates increased autophagy activity [19,29,30]. We compared the changes in the level of these proteins in the small bowel grafts by means of immunohistochemistry. Intestinal tissues fixed in 10% neutral buffered formalin and embedded in paraffin were cut in serial 3 μm thick sections. After deparaffinization and rehydration, samples were pretreated with heat induced epitope retrieval method in 1 mM (pH = 6.0) citrate buffer (Histopathology Ltd., Pécs, Hungary) in a microwave oven for 15 min at 750 W. After cooling at room temperature, tissues were washed in TRIS buffered saline solution (TBS) (pH = 7.6). For immunohistochemistry, samples were incubated in Beclin-1 antibody (Cat. Nr. Bs-1353R, Bioss Antibodies Inc., Woburn, MA, USA 1:2000, 1 h at room temperature), LC3B antibody (Novusbio NB100-2220, 1:400), and p62/SQSTMI(Cat Nr. p0067. Sigma-Aldrich Ltd., St. Louis, MO, USA 1:2000, 1 h at room temperature). Sections were washed in TBS and incubated with HISTOLS-R anti-rabbit HRP labelled detection system (Cat. Nr. 30011.R500, Histopathology Ltd., Pécs, Hungary, 30 min at room temperature,). After washing in TBS, the reaction was developed with HISTOLS Resistant AEC Chromogen/substrate System (Cat. Nr. 30015, Histopathology Ltd., Pécs, Hungary), while controlling the intensity of the staining under microscope. Sections were counterstained with hematoxylin solution, and bluing was performed with tap water. Samples were dehydrated in alcohol, cleared in xylene, and mounted with permanent mounting medium. Slides were digitized with a Mirax scanner and photographs were taken with CaseViewer 2.4 software (3DHISTECH Ltd., Budapest, Hungary).

Analysis of stained tissues for the respective proteins was performed with the help of the IHC profiler plug-in of the Image J software, and the optical density (OD) was scored according to the method previously described [31]. The image processing software showed a percentage of stained areas in the slide as High Positive (HP), Positive (P); Low Positive (LP); and Negative (N). From these numerical values, the Optical density score is calculated using the recommended algebraic formula: (HP × 4 +P × 3 +LP × 2 +N × 1)/100.

### 2.9. Statistical Analysis

For statistical evaluation, one-way analysis of variance (ANOVA) was used, followed by adequate post-hoc tests for multiple comparisons. The Kruskal–Wallis test was used for analysis of histological and IHC scores. Comparing changes within a group was performed using the paired *t*-test. All data are represented as the mean ± SEM, unless otherwise stated. The difference was considered statistically significant when *p* value was less than 0.05.

## 3. Results

### 3.1. Histology

#### 3.1.1. Morphology

The histology slides were analyzed blindly by two individuals. The microscopic changes were described using the scoring system described in Table 1. The injury scores are hereby denoted as histological injury score (HIS).

There was no significant microscopical injury to the intestinal mucosa in the sham group. When observed, the bowel architecture in these samples was maintained (HIS of 0).

After perfusion of the experimental groups (PC, Rapa-0, Rapa-30, and Rapa-60), minimal mucosa injury could be observed. Mostly, a few villi belonging to these groups displayed subepithelial spaces at their tips (HIS of 1). In the PC group and Rapa-0 group, there was also infrequent occurrence of villus denudation (HIS of 4). Nonetheless, the mean histological injury present in each experimental group was statistically insignificant when compared to the sham group.

**Figure 1:**
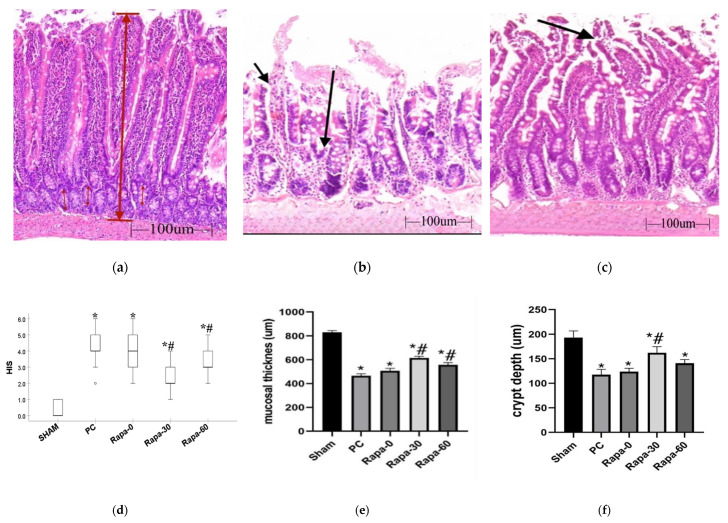
Histology and Morphometric measurements after cold preservation. (**a**–**c**) are representative images after HE staining for sham (**a**); PC (**b**) and Rapa-30 (**c**), respectively. (**a**) represents normal intestinal mucosa. This figure is annotated to illustrate how the mucosal thickness (thick, long red arrow) and crypt depth (thin, short red arrow) were measured in our samples. (**b**) shows severely damaged mucosa. Short arrow represents complete epithelial breakdown around villus, while long arrow represents total villus loss; both of which are characteristic for the PC group. (**c**) represents the characteristic intestinal injury in the Rapa-30 group. The arrow points to subepithelial space extending from the villus tip to the body of the villus. (**d**) is a representative dot plot for HIS scores of the various groups after preservation. *n* = 6. Data are shown as median (interquartile range); * *p* < 0.0001 vs. sham. # *p* < 0.05 versus PC group. (**e**,**f**) are graphical representation of mucosal thickness and crypt depth measurement respectively. *n* = 6. Data are mean ± SEM; * *p* < 0.05 vs. sham. # *p* < 0.05 versus PC group.

After 12 h of cold ischemia, there was exacerbation of the mucosa injury in the experimental groups. Intestinal mucosa injury was significantly worse in these groups (PC, Rapa-0, Rapa-30, and Rapa-60) compared to the sham (*p* < 0.0001). This indicates that cold preservation causes significant mucosa damage. With regard to the PC group, villi here were frequently denuded, with its core exposed. There was complete loss of villous tissue in some areas, and moderate crypt layer destruction (Median HIS = 4; interquartile range: 4–5). Among the experimental groups, the Rapa-30 grafts showed the most significantly attenuated mucosa injury compared to the PC group (*p* < 0.0001). The mucosa here was characterized mostly by subepithelial spaces at the upper half of the villus, these spaces sometimes extended down the side. Infrequently, some villus denudation was recorded (Median HIS = 2; interquartile range: 2–3). The Rapa-60 group also displayed significantly better mucosa structure compared to the PC (*p* = 0.0093), albeit to a lesser degree than Rapa-30. Mucosa belonging to this group showed frequent areas of epithelial lifting, epithelial breakdown, and a few denuded villi (Median HIS = 3; interquartile range: 3–4). Even though the Rapa-0 group seemed less damaged than the PC group, this injury was not significantly different from the PC (*p* = 0.1523). Mucosa here also displayed crypt injury, villi denudation, and loss (Median HIS = 4; interquartile range: 3–5). (Figure 1a–d).

After reperfusion, there was significant difference in all experimental groups when each was compared to the end of their individual storage period (*p* < 0.05). When compared to the PC group, the Rapa-30 group once again showed less mucosa damage (*p* = 0.0001). Grafts here were mostly characterized by epithelial lifting down the sides of the villi. In addition, we observed some regions with missing epithelial covering at villi tips, sometimes extending to half of the villi (Median HIS = 4; interquartile range: 3–5). The Rapa-60 mucosa damage was still significantly less than the PC (*p* = 0.0237). The villi of this group were mostly denudated, and some regions showed loss of villi component, with minimal crypt layer damage (Median HIS = 5; interquartile range: 4–5). The Rapa-0 and PC group showed no significant difference at the end of reperfusion. Both groups showed massive villi loss and crypt damage (Median HIS for PC = 6; interquartile range: 5–6; Median HIS for Rapa-0 = 5; interquartile range: 5–6).

#### 3.1.2. Morphometry

After 12 h of cold ischemia, we also performed morphometric analysis of the intestinal mucosa. Mucosa thickness decreased significantly in all groups compared to the sham operated group (*p* < 0.0001). Compared to the PC group, the decrease in mucosa length was significantly attenuated in the Rapa-30 group (*p* < 0.0001) and the Rapa-60 group (*p* = 0.0014). In comparison, the PC group and the Rapa-0 group had no significant difference (*p* = 0.2339) (Figure 1e).

Analysis of the crypt depth revealed that compared to the PC group, only the Rapa-30 showed a significant attenuation of crypt depth decline (*p* = 0.0064). Though the measured values for Rapa-0 and Rapa-60 showed slight attenuation of the crypt depth, these values did not reach statistical significance when compared to the PC group (*p* = 0.9428 and *p* = 0.2352, respectively) (Figure 1f).

The summary of the histology results suggests that Rapamycin is an effective preconditioning agent capable of attenuating mucosa ischemia and reperfusion injury, especially if administered 30 min before organ retrieval.

### 3.2. Biochemical Analysis

**Figure 2:**
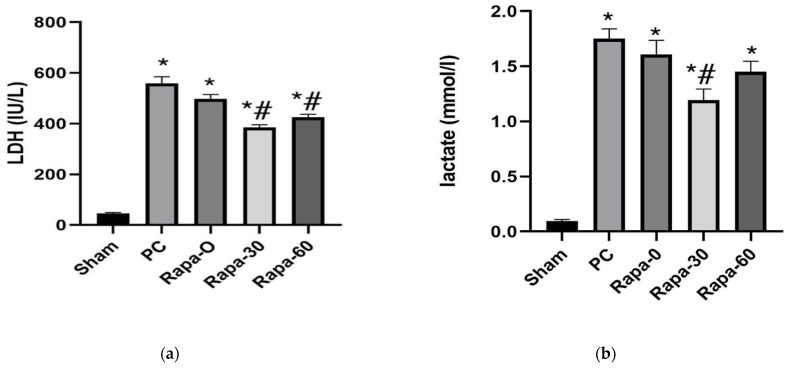
Graphical representation of changes in LDH (**a**) and lactate (**b**) after cold preservation. Data are mean ± SEM. *N* = 6. * *p* < 0.0001 vs. sham. # *p* < 0.05 versus PC group.

After 12 h of preservation, intestines of the Rapa-30 and the Rapa-60 showed significantly less release of LDH than those of the PC group (*p* < 0.0001 and *p* = 0.0024, respectively). In comparison, there was no significant difference in the level of the LDH marker between the PC group and the Rapa-0 group (*p* = 0.3524). (Figure 2a).

Similarly, after 12 h of storage, the Rapa-30 group still showed significantly less release of lactate compared to the PC-group (*p* = 0.0002). The Rapa-60 group also showed a tendency for the lactate content in the preservation fluid to be less compared to the PC grafts. However, this value did not decrease to our set level of statistical significance (*p* = 0.0797). Grafts belonging to the Rapa-0 and those belonging to the PC group showed similar levels of lactate (*p* = 0.6461) (Figure 2b).

Taken together, the results from the cell injury markers confirm that intestinal preservation injury can be attenuated by Rapamycin, especially when administered 30 min before organ retrieval.

### 3.3. IHC (Autophagy Proteins)

By immunohistochemistry staining methods, we preliminarily evaluated markers of autophagy.

First, we determined what changes cold preservation had on autophagy in the intestine grafts by comparing the sham group and the Preservation group (PC). At the end of preservation, the PC group showed decrease in LC3B (*p* < 0.001) and Beclin-1 (*p* < 0.001) proteins compared to the sham. In this group, we also observed an increase in the p62 protein (*p* < 0.001) compared to the sham. Taken together, these results suggest a suppression of autophagy in the intestine after cold preservation.

Next, we compared the Rapamycin treated groups to the PC group to determine if the drug could upregulate autophagy after cold preservation. At the end of cold storage, the experimental groups that were pretreated with Rapamycin at 30 min (Rapa-30) and 60 min (Rapa-60), respectively, had a significant increase in autophagy activity compared to the PC group. In these groups, there was a significant increase in the LC3B protein compared to the PC group (*p* < 0.001 and *p* = 0.007, respectively). Similarly, a significant increase in the Beclin-1 protein was observed (*p* < 0.001 and *p* = 0.008, respectively). In contrast, the p62 protein was significantly reduced in both groups compared to the PC (*p* < 0.001 and *p* = 0.005, respectively).

There was no significant difference in autophagy activity between the Rapa-0 group and the PC group. Even though the LC3B and Beclin-1 proteins appeared to increase in the Rapa-0 group, these values were statistically insignificant (*p* = 0.139 and *p* = 0.290, respectively). The p62 protein in the Rapa-0 group slightly but insignificantly decreased compared to the PC group (*p* = 0.239).

**Figure 3:**
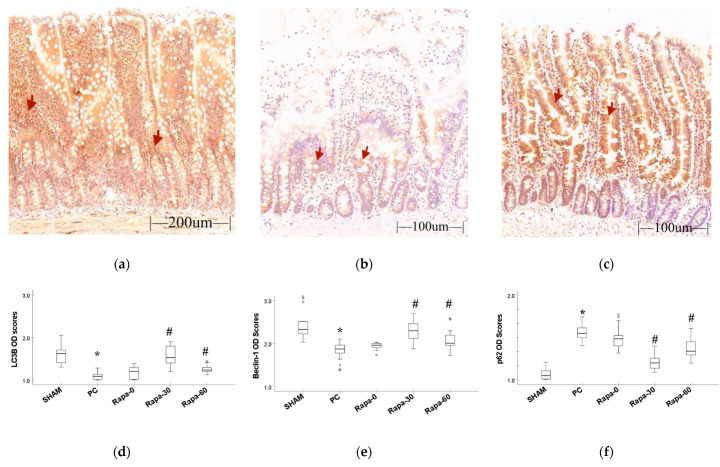
Immunohistochemistry result. (**a**–**c**) represent staining for LC3B protein for sham, PC, and Rapa-30 groups, respectively. This method stains the cytoplasm and certain elements in the lamina propria brown (red arrows are examples of immunoreactive areas). (**d**–**f**) are graphical representation for changes in the IHC optical density scores for LC3B, Beclin-1, and p62 proteins, respectively. Data are median and interquartile range. *n* = 6. * *p* ≤ 0.001 for PC vs. sham. # *p* ≤ 0.05 versus PC. (Representative pictures for Beclin-1 and p62 staining can be found in Supplementary Material Figure S1).

Taken together, these results suggest that Rapamycin, especially when administered at 30 min, promotes autophagy in the intestinal mucosa (Figure 3).

## 4. Discussion

This present study investigated the anti-ischemic effect of donor pretreatment with a single dose (2 mg/kg) of Rapamycin and correlated it with the time of drug administration in a model of small bowel perfusion and preservation. Ischemic injury is defined by mucosa changes and cell markers of injury (LDH and lactate).

Organ retrieval and preservation exposes the tissues to ischemic damage [2]. With regard to the intestinal graft, this usually manifests as extensive mucosa damage [2,8,32]. In our study, we also observed that by the end of the preservation period (12 h), all the experimental groups exhibited significant mucosa and cellular injury. This we interpreted as a confirmation that ischemic damage occurred in our model of intestinal cold preservation.

Reducing ischemia-reperfusion damage by applying ischemic or pharmacological pretreatment techniques has been described in various clinical trials [33,34], and experimental organ transplantation [14,35]. In this study, we preconditioned experimental animals with Rapamycin at different time points. Our results describe a tendency for Rapamycin protection against the cold preservation ischemia of the small bowel grafts. This protective effect was significantly observed when the drug was administered at least 30 min before organ retrieval (as seen in the Rapa-30 and Rapa-60 groups). In addition, even though remarkable mucosa damage happened in both groups upon reperfusion, the injury was not as severe as the PC group. We believe that this is because the mucosa of both the Rapa-30 and Rapa-60 intestinal grafts showed lesser damage by the end of the cold storage. Therefore, the resulting injury in the reperfusion phase was minimal when compared. This is in line with other studies that have shown that the extent of intestinal ischemia/ischemic damage largely determines the magnitude of the reperfusion injury [2,8]. Taken together, our results indicate that pretreatment with Rapamycin can attenuate the cold ischemia and reperfusion injury of the intestine.

Rapamycin is a known activator of autophagy [19,30,36]. The autophagy pathway, which is necessary for functional homeostasis and cell survival, has been implicated in intestinal ischemia-reperfusion injury [19,37]. Therefore, we preliminarily investigated changes in the autophagy pathway as a way of determining the possible intrinsic effects of Rapamycin preconditioning on small bowel grafts. Many studies monitor this pathway by using Beclin-1 and LC3B, key proteins involved in its initiation and elongation, and p62, a protein degraded by this pathway [19,29,30]. Consequently, we also adopted these proteins in our investigations.

Autophagy proteins LC3B and Beclin-1 decreased while p62 increased in our preservation control (PC) group compared to the sham. This is usually interpreted as impaired autophagy activity [30]. This pattern of autophagy in our PC group is not the norm in ischemic tissues. Ischemia tends to upregulate the autophagy machinery [19,22]. However, studies have shown that extended periods of ischemia can also impair the autophagy pathway [24,38,39]. Why the autophagy mechanism is sometimes dysregulated during ischemia is unclear. It might be that prolonged starvation causes depletion of essential autophagic components and inhibition of its key regulators, such as Beclin-1 [39]. We didn’t explore the exact reason for this occurrence in our results. However, following the administration of Rapamycin, autophagy proteins LC3B and Beclin-1 increased, while p62 decreased compared to the PC group. This was especially the case in the Rapa-30 and the Rapa-60 groups. Our results therefore indicate that Rapamycin enhanced autophagy in the cold preserved small bowel grafts. These findings agree with previous studies which show that Rapamycin stimulates and sustains autophagy response in various stress conditions [30,36].

Analyzing the results from both the preservation injury and autophagy protein experiments together, a pattern becomes evident: samples with enhanced autophagy also had a better-preserved mucosa structure. Based on this, we suggest that Rapamycin exerts its effect during small bowel preservation, at least in part, by inducing cytoprotective autophagy in a timely manner. This interpretation is guided by similar ischemia-reperfusion studies, which have shown Rapamycin acting through autophagy to attenuate ischemic damage in different organs [18,24,29]. Moreover, this possibility is further supported by studies that have associated induced autophagy with better preserved intestinal mucosa barrier [25,37,40].

In our study, we noted a time-dependence in Rapamycin’s actions. The effects of administering the drug 30 min prior to cold intestinal ischemia was superior to giving the drug at 60 min prior. We do not think that the duration of drug action can fully explain this difference. This is because Rapamycin is a fast and long-acting drug with a half-life lasting over 24 h [41]. A more plausible explanation might lie in the inhibitory effect Rapamycin has on the mTOR pathway. Rapamycin’s modulation of the mTOR is responsible for most of its effect on metabolism, including the upregulation of autophagy [16,19]. Studies have shown a time and dose dependence of Rapamycin’s administration on mTOR activity [41,42]. In this present study, the Rapa-30 group showed a stronger increase in autophagy activity, which might imply greater mTOR inhibition. Hence, we are tempted to speculate that an effective therapeutic window for administering Rapamycin prior to small bowel cold ischemia exists. Within this timeframe, Rapamycin can effectively inhibit mTOR, thus activating the autophagy machinery and other downstream effects. However, such a conclusion can only be drawn after more extensive time- and dose-dependent studies and by making use of more biological methods to characterize the mTOR and autophagy pathways. This can be a future research direction.

Our study has some limitations. We have only described changes in bowel mucosa after cold preservation but not transplant grafts. Due to this, any effect Rapamycin may have on the rat’s blood and hemorheological parameters, as well as survival rates, cannot be determined. Additionally, in this study we have only focused on Rapamycin acting through the autophagy pathway. However, we would like to highlight that there might be other mechanisms for Rapamycin’s effects. For example, Rapamycin has been shown to modulate the expression of myosin light chain (MLC), an important contractile protein [43]. Regulating the expression and phosphorylation of this protein might play a role in ongoing intestinal mucosa damage [44,45]. Additionally, Rapamycin can induce certain antioxidant proteins [43]. Studies have shown that improving the antioxidant capacity of the small bowel graft correlates with reduced preservation-reperfusion injury [14,32]. Consequently, future studies would be necessary to explore the effect of Rapamycin at more time points during the process of small bowel transplantation and to establish a wider spectrum of its mechanism of action.

In summary, our study has demonstrated a protective effect of Rapamycin pretreatment on small bowel grafts during cold preservation. We have observed that this protection is dependent on the time the drug was administered before organ retrieval. We have also suggested the autophagy pathway as being key to Rapamycin’s effect. Indeed, Rapamycin has been previously used in the field of small bowel transplant research, but these other studies were centered around its immunosuppressive function and post-implant survival effect [46,47]. However, we have rather narrowed our interest to its mucosa protective effect during cold preservation, thereby suggesting Rapamycin as a possible addition to intestinal preconditioning protocols or preservation solutions.

## Figures and Tables

**Table 1:** Intestinal Histology Injury Scores (HIS) as described by Park et al. [8].

Injury Score	Description
0	Normal mucosa
1	Subepithelial space at the tips of the villi
2	Extension of the epithelial spaces
3	Massive epithelial lifting down the sides of the villi
4	Denudation of the villi
5	Loss of villi
6	Crypt layer damage
7	Transmucosa infarction
8	Transmural infarction

## Data Availability

Data are available in the text and upon request from the corresponding author.

## Supplementary Materials

The following supporting information can be downloaded at: https://www.mdpi.com/article/10.3390/metabo12090794/s1, Figure S1: Immunohistochemistry staining for autophagy proteins (Beclin-1 and p62) at the end of cold preservation.
