# Supplementary material for: The Effects of Rapamycin on the Intestinal Graft in a Rat Model of Cold Ischemia Perfusion and Preservation
Source: Metabolites. 2022 Aug 25;12(9):794. doi: 10.3390/metabo12090794 (PMC9505496; doi:10.3390/metabo12090794)
Supplement: Supplementary file 1 [file metabolites-12-00794-s001.zip › metabolites-1826571-supplementary.pdf]

Supplementary Materials

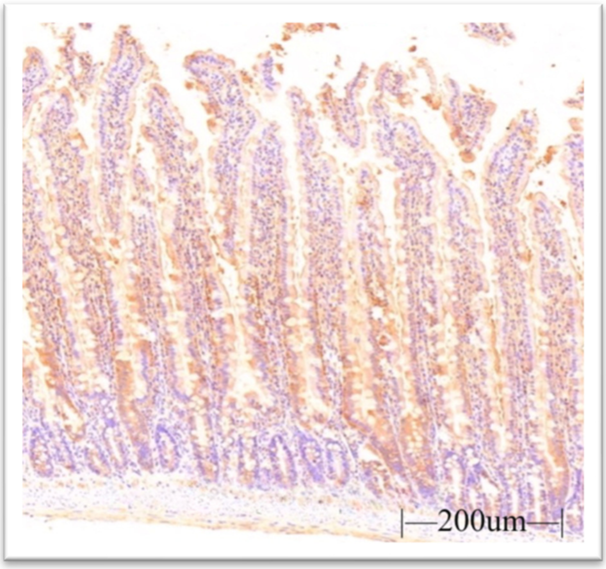

(a)

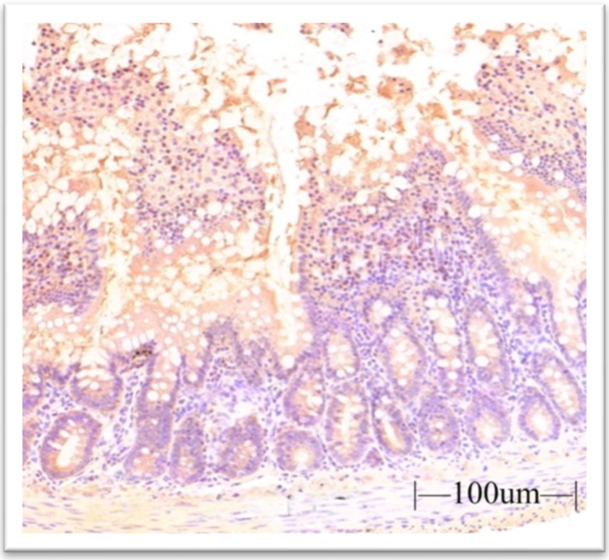

(b)

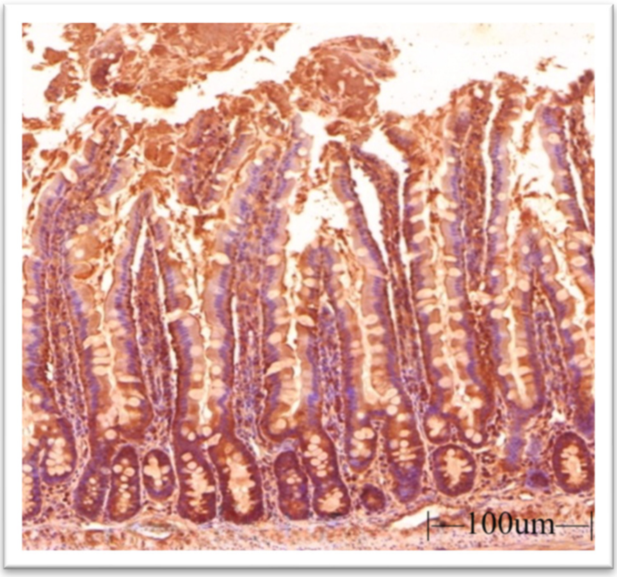

(c)

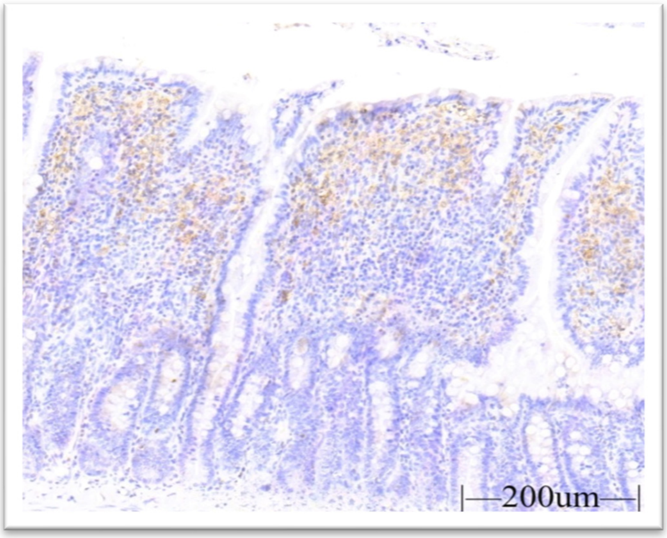

(d)

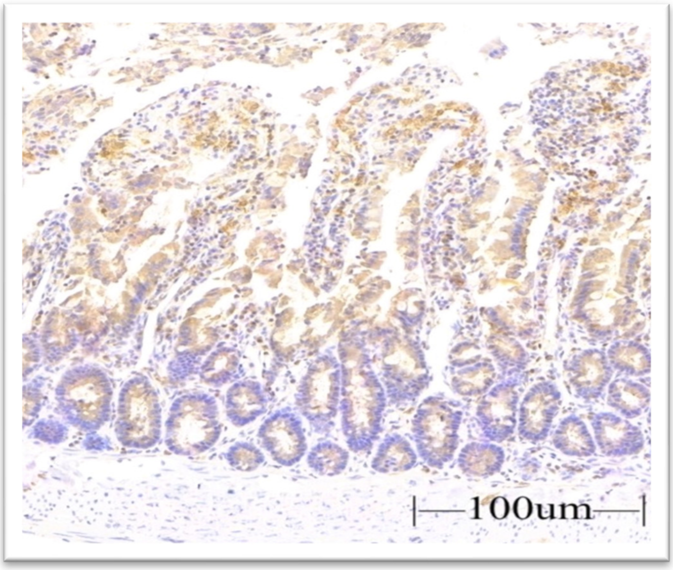

(e)

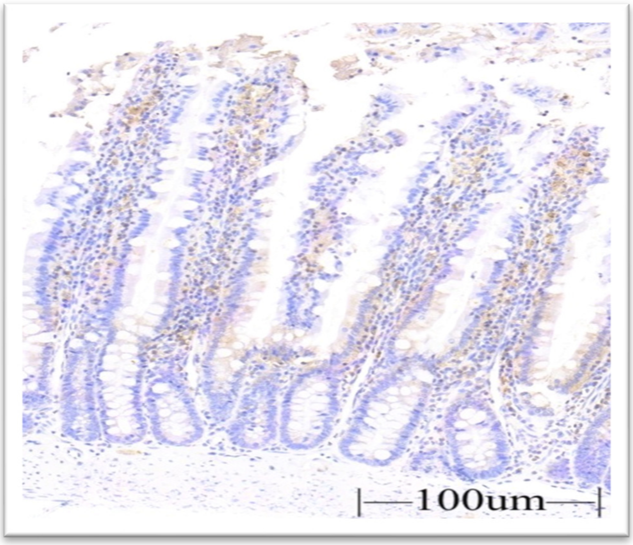

(f)

Figure S1. Immunohistochemistry staining for autophagy proteins (Beclin-1 and p62) at the end of cold preservation. (**a**–**c**) Changes in autophagy proteins. Panels a-c represent changes in the groups for the Beclin-1 protein in the following order: Sham, PC, and Rapa-30 respectively. Brown staining of the cytoplasm and certain elements in the lamina propria is considered positive staining for this protein. The more intense the staining, the greater the presence of Beclin-1 in the sample. (**d**–**f**) Changes in autophagy proteins. Panels a-c represent changes in the groups for the p62/SQSTMI protein in the following order: Sham, PC, and Rapa-30 respectively. Brown staining of the cytoplasm and certain elements in the lamina propria is considered positive staining for this protein. The more intense the staining, the greater the presence of p62 in the sample.
